# Epidemiologic and Environmental Investigations of Reported Hantavirus Cases Inform Exposure Risk in California, 1993–2020

**DOI:** 10.4269/ajtmh.25-0270

**Published:** 2025-10-16

**Authors:** Bryan T. Jackson, Anne M. Kjemtrup, Mark G. Novak, Curtis L. Fritz, Sharon Messenger, Mojgan Deldari, Joseph E. Burns, Duc J. Vugia, Vicki L. Kramer

**Affiliations:** ^1^ Infectious Diseases Branch, California Department of Public Health, Sacramento, California;; ^2^ Viral and Rickettsial Disease Laboratory, California Department of Public Health, Richmond, California

## Abstract

From 1993 to 2020, 89 hantavirus cases were reported in California residents. Fifty-six (63%) were male, mean age was 41.5 years old, and 28 (31%) of the cases were fatal. The majority of cases were classified as hantavirus pulmonary syndrome, and infections peaked during summer and spring. A total of 70 environmental investigations were conducted for 77 cases (87%). Visual evidence of rodent activity was detected in 52 (74%) of the investigations, and 36 patients (40%) reported direct contact with rodents or having cleaned rodent-affected areas. In total, 1,353 rodents (73%) captured during the investigations were *Peromyscus maniculatus*, and 264 (20%) tested positive for Sin Nombre virus (SNV) antibodies. Probable exposure locations were identified for 81 cases (91%), with the majority of exposures occurring in the patient’s county of residence. Indoor exposure was most common, and more exposures occurred in peridomestic environments than at worksites or recreational areas. Epidemiological and environmental investigations and SNV sequencing were crucial in identifying exposure sites. Enhanced awareness and preventive measures, especially in high-risk areas and among at-risk populations, are essential to mitigate future cases.

## INTRODUCTION

In 1993, an outbreak of acute respiratory disease occurred in the Four Corners region of the United States.^1^^,^^2^ Investigation by public health officials identified a previously unrecognized hantavirus (RNA virus) as the etiologic agent, which was provisionally named Sin Nombre virus (SNV)^1^ and has recently been reclassified as *Orthohantavirus sinnombreense*.^3^ Deer mice (*Peromyscus maniculatus*) were identified as the primary reservoir in the western United States.^4^

Deer mice maintain SNV for an indefinite period and periodically shed SNV in their excreta.^5^ Humans become infected through inhalation of aerosolized infectious rodent excreta.^6^ Rarely, humans may also be infected by rodent bites and accidental virus ingestion of contaminated food.^5^^–^^7^ Activities such as cleaning (e.g., sweeping, vacuuming, and rodent trapping) that aerosolize the virus in enclosed structures can increase the risk of infection in areas where deer mice are present.^7^^–^^9^

The clinical presentation of hantavirus pulmonary syndrome (HPS) described by Hughes et al.^10^ includes a febrile prodrome and gastrointestinal complaints followed by the abrupt onset of pulmonary edema as well as cardiac insufficiency leading to death. Most HPS patients exhibit an elevated hematocrit, hypoalbuminemia, and thrombocytopenia.^11^ Hantavirus pulmonary syndrome fatality rates can be as high as 35–50%.^12^ It was subsequently recognized that people infected with SNV may also develop a nonpulmonary hantavirus infection, where symptoms include the febrile prodrome with no or only mild respiratory deficits.^13^

Cases of HPS were identified in the western United States, including California,^14^ in the years after the initial outbreak. Hantavirus pulmonary syndrome was made nationally notifiable in 1995, and the Council of State and Territorial Epidemiologists (CSTE) amended the surveillance case definition to include nonpulmonary SNV infections in 2015.^15^ In California, 0–7 sporadic cases were reported each year since 1993,^16^ with a single outbreak of 10 cases from Yosemite National Park, including eight California residents, in 2012.^17^

Although deer mice are found statewide^18^ and SNV-reactive antibodies have been detected in deer mice from 47 of 58 California counties,^19^ hantavirus cases in the state are typically associated with exposure in rural and mountainous areas.

The California Department of Public Health (CDPH) Vector-Borne Disease Section (VBDS) attempts to investigate all hantavirus cases that may have been acquired in the state and performs both case-based and general rodent surveillance for SNV detection to document locations and activities with increased human risk.

Notable California hantavirus cases and related environmental surveillance findings have been previously described^14^^,^^17^^,^^20^^–^^25^; however, given the ever-changing ecology of viruses and their vertebrate hosts, an updated comprehensive summary of reported hantavirus cases in California residents is warranted. The objectives of this paper were to summarize the key findings from investigations of reported hantavirus cases from 1993 to 2020 and demonstrate which types of information (epidemiologic, environmental, or molecular SNV testing) were most valuable to identify likely exposure locations. Information from this summary may guide future public health response to hantavirus cases.

## MATERIALS AND METHODS

Hantavirus cases were identified through disease reporting to the CDPH in accordance with California Code of Regulations Title 17, Section 2500. Serologic testing of a sera bank maintained at the CDPH Viral and Rickettsial Disease Laboratory from acute respiratory distress syndrome (ARDS) cases with no identified etiologic agent was used to retrospectively identify several cases among California patients who were ill or died before 1993. All California residents who met criteria for a confirmed case as outlined in the CSTE surveillance case definition were included in this study. As stated in the case definition and previously described,^25^ laboratory confirmation included serology, reverse transcriptase polymerase chain reaction, and sequencing when sample volume was sufficient.

State and local public health officials endeavored to identify locations where and circumstances under which patients were infected through interviews with the patients and/or proxies (e.g., family members) using standardized forms in the California reporting system, which include questions pertaining to travel, rodent exposure, and symptom onset.^26^ Based on responses to questions dealing with visualization of rodents or signs of rodents, state and/or local public health officials conducted visual inspection of putative exposure locations and when possible, rodent trapping to confirm the presence of deer mice.

Visual inspections consisted of exterior and interior walk-throughs of a structure or possible exposure location when applicable. Rodent entry points, rodent feces/urine, rub/gnaw marks, dead/live rodents, rodent traps, and rodent nests along with any other indicators of rodents were noted. Locations with evidence of rodents were an area of focus for rodent trapping.

Rodent trapping typically consisted of setting baited Sherman live traps in the afternoon and collecting them the next morning. Captured rodents were processed according to CDPH animal care use protocols. Species identifications were determined through a combination of morphological characteristics and measurement data (head and body length, tail length, weight, ear length, and foot length). When uncertain of species identification, we consulted the Museum of Vertebrate Zoology, University of California, Berkeley for species confirmation. Rodent trapping and processing were performed as previously described.^20^^,^^27^

Human and rodent sera were tested for antibodies directed against the SNV nucleocapsid (N) protein by ELISA using methods previously described.^28^^–^^30^ Human sera were screened for both SNV-specific IgM and IgG antibodies, whereas rodent sera were tested for only SNV-specific IgG antibodies. Specimens detecting N protein antibody at a dilution of >1:400 were considered positive.^4^

RNA from human serum samples or rodent blood was extracted using the QIAamp Viral RNA Mini Kit (catalog no. 52906; Qiagen, Venlo, Netherlands) and the Qiagen QIAamp RNA Blood Mini Kit (catalog no. 52304), respectively; then it was tested for SNV using a modified real-time reverse transcription polymerase chain reaction (rRT-PCR) protocol as previously described,^31^ targeting an 82-base fragment of the S segment. In this modified protocol, the Invitrogen™ SuperScript™ III Platinum™ One-Step qRT-PCR Kit (catalog no. 11732-088; Thermo Fisher Scientific, Carlsbad, CA) was used (5 *µ*L extracted RNA in a 25-*µ*L total reaction volume). Cycling conditions were adjusted to 1) 50°C for 30 minutes, 2) 95°C for 2 minutes, and 3) 45 cycles of 95°C for 15 seconds and 60°C for 1 minute.

Genetic sequences generated at CDPH from patients were compared with sequences from California rodents collected during routine rodent surveillance and disease case investigations. Sin Nombre virus RNA samples positive by rRT-PCR were genotyped by Sanger sequencing a 940-base pair fragment (nucleotides 22–962) of the glycoprotein precursor (*GPC*) on the medium segment of the SNV genome using primers M1L and M962R published in Bagamian et al.^31^ Phylogenetic analyses using maximum likelihood and Bayesian models were performed to reconstruct trees from sequences generated at CDPH as well as available SNV sequence data on GenBank as previously described.^25^

To summarize the geographic exposure locations and the types of information used to make these determinations, we categorized the exposure locations as county of residence, outside county of residence, or undetermined. Likely exposure locations and circumstances were determined by considering the available information obtained from interviews and investigations (i.e., evidence of rodent infestation or confirmation of deer mice at a patient’s residence, work, or recreational site). When available, SNV sequences from patients were compared with SNV sequences obtained from deer mice captured during the investigations or during routine statewide surveillance to help identify, support, or refute a region of likely exposure. The most probable location where each patient was infected was designated by the preponderance of epidemiologic, environmental, and molecular information.

Human case statistical analysis was performed using Epi-Info^™^ (CDC, Atlanta, GA) and Excel (Microsoft Corp, Redmond, WA). Statistical analyses were univariate comparisons using risk ratios, *t*-tests, Fisher exact tests (considering two-tailed *P*-values), or χ^2^ statistics as appropriate. Incidences per 100,000 population were estimated using the number of cases in a defined time period divided by the average population in the state or county of interest in that period as reported by the California Department of Finance.^32^

Statistical relationships between human cases and elevation were examined using R v. 3.5.0 (R Foundation, Vienna, Austria). ArcGIS Pro v. 3.0.0 (Esri, Redlands, CA) was used to obtain estimates for land area (kilometers squared) by elevation using digital elevation model data for California (90-m resolution).^33^ We simplified human cases into the presence of at least one hantavirus human case (“occurrence”) to eliminate the potential confounding effect of having multiple human cases associated with the same location and time. We examined potential nonlinearity in the relationship between case occurrence and elevation by comparing linear and polynomial models. Akaike information criteria values were used to select the best fitting among competing models (Supplemental Tables 1 and 2).

## RESULTS

### Demographics and epidemiologic summary.

**Figure 1.**
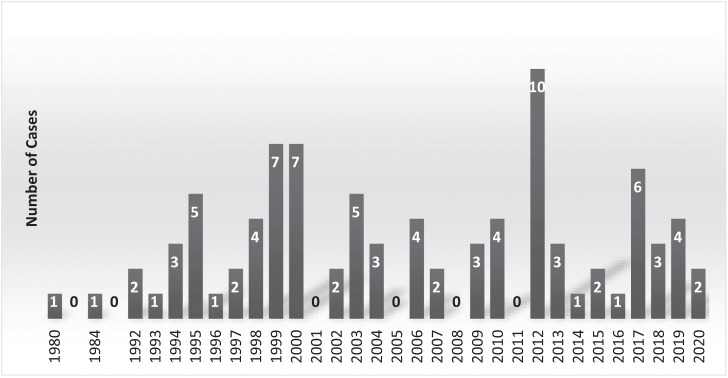
Hantavirus cases reported by year in California from 1993 to 2020, including four cases from 1980 to 1992 that were retrospectively identified.

From 1993 to 2020, 89 cases of hantavirus infection were reported in California residents (median per year: 3; range: 0–10) (Figure 1). Four cases occurred before identification of SNV in 1993 (1980–1992) and were retrospectively identified from ARDS sera collection. Seventy-six cases were attributed to exposure in a California county, whereas exposure circumstances were outside of California and undetermined for 5 and 8 cases, respectively.

**Figure 2.**
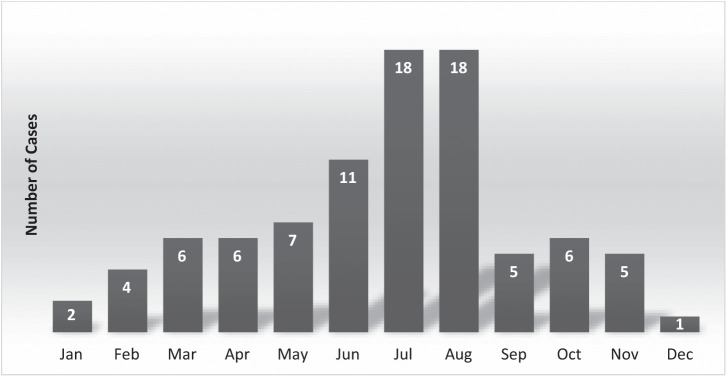
Hantavirus cases by reported month of onset for 89 California residents from 1993 to 2020.

Fifty-six patients (63%) were male, and the mean age of patients was 41.5 years old (range: 12–78 years old). A fatal outcome was reported for 28 cases (31%). There was no statistical association (*P* >0.05) between age, gender, and outcome (fatal or nonfatal). Eighty-three cases (93%) were classified as HPS, and 6 cases (7%) were classified as nonpulmonary syndrome, 4 of which were from before the 2015 National Notifiable Diseases Surveillance System case definition change. Months of onset were most commonly summer (June through August: 47) and spring (March through May: 19) months followed by autumn (September through November: 15) and winter (December through February: 8) months (Figure 2).

Thirty-five patients or their proxies (39%) reported observing evidence of recent rodent activity at their residence (including garages and outbuildings), whereas 12 (13%) reported recent rodent activity at their worksites. An additional five patients or proxies (6%) reported evidence at both their residences and worksites. The remaining 37 patients or proxies (42%) either reported no rodent activity or gave no answer.

Thirty-six patients (40%) reported that in the 2 months before onset, they had contact with rodents or had cleaned (e.g., swept, vacuumed, or moved/packed stored materials) an enclosed space where evidence of rodent activity was noted by the patient, a proxy, or public health officials during the subsequent case investigation. An additional nine patients (10%) reported performing similar cleaning actions where no evidence of rodent activity was observed.

### Environmental investigations.

A total of 70 environmental investigations were conducted for 77 (87%) of 89 cases. Two of these investigations included multiple cases, totaling nine cases, associated with the same exposure locations. Two cases with out-of-state exposures had environmental investigations conducted by environmental officials in their respective states of exposure (New Mexico and Utah). Case investigations were not conducted for 12 cases because the patient or proxy declined to provide minimum essential information or access to possible exposure locations. The median number of days between illness onset and the start of the environmental investigation was 26 days (range: 5–555 days).

Environmental investigations were conducted at 137 locations identified through information obtained from patient interviews. Locations included 59 patients’ residences (43%) and 31 workplaces (23%) as well as 19 recreational areas (14%) and 28 other locations (20%). Visual evidence of rodent activity (e.g., feces, chew marks, nest, etc.) was detected during 52 (74%) of 70 investigations.

Live rodent trapping was conducted as part of 63 (90%) of 70 environmental investigations. The median number of trap nights per investigation was 100 (range: 10–1,500 trap nights). At least one *P. maniculatus *was captured during 60 (95%) of 63 investigations that included rodent trapping. A total of 1,353 *P. maniculatus* were captured, comprising 73% of all rodents collected. The median number of *P. maniculatus* captured per surveillance event was 12 (mean: 20; range: 0–126). Of 576 *P. maniculatus* captures with specific trap location recorded (i.e., indoors versus outdoors), 533 (93%) were captured outdoors. Age was recorded for 1,234 *P. maniculatus*, with adults making up 78% (960).

Forty-nine (78%) of the environmental investigations with rodent trapping had at least one species of rodent other than *P. maniculatus* collected. Of the 538 rodents making up other species, 85 (16%) were *Neotoma* spp., 83 (15%) were *Peromyscus boylii*, 68 (13%) were *Peromyscus truei*, 38 (7%) were *Microtus* spp., 22 (4%) were *Peromyscus californicus*, and the remaining 242 (45%) were other species or not identified.

Two hundred and sixty-four (20%) of 1,346 *P. maniculatus* blood samples tested positive for SNV antibodies, significantly higher (χ^2^ [2, *N* = 16,200] = 98.60, *P* < 0.01) than the 11% seroprevalence statewide for surveillance not associated with a case investigation. Forty-four (70%) of the environmental investigations with rodent trapping yielded at least one seropositive *P. maniculatus*. Of 233 seropositive *P. maniculatus *identified to age, 211 (91%) were adults. Sin Nombre virus-reactive antibodies were detected in 14 (3%) of 473 other rodent species (4 *Microtus californicus*, 3 *P. truei*, 2 *Neotoma lepida*, 1 *Microtus montanus*, 1 *Microtus* spp., 1 *Perognathus parvus*, 1 *Reithrodontomys megalotis*, and 1 *Neotamias minimus*).

**Figure 3.**
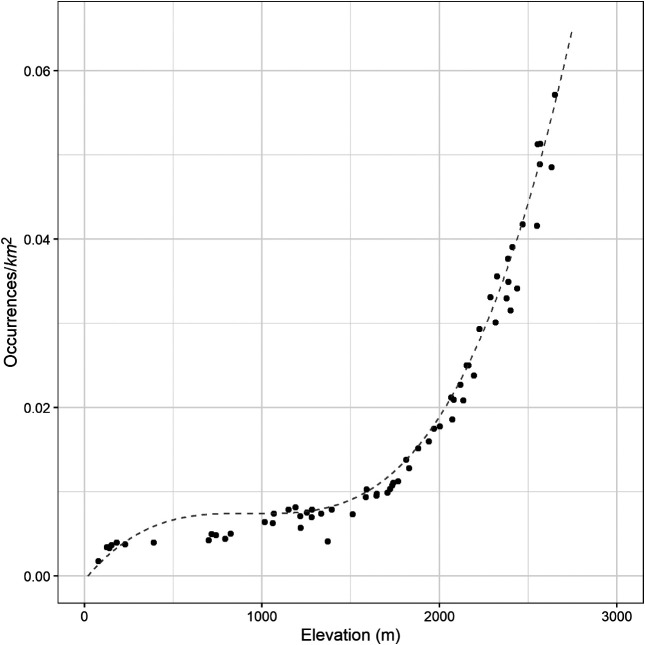
Polynomial regression (order 3) between elevation and hantavirus occurrences per kilometer squared for the likely exposure location for California residents. A total of 65 elevations from 70 exposure locations were included in the graph.

The median elevation of all sites surveyed was 1,734 m (range: 79–2,652 m). The risk of hantavirus infection was positively associated with elevation (Figure 3; Supplemental Figure 1). Both the number of human cases per area (Supplemental Figure 1) (*R*^2^ = 0.85, *P* < 0.001) and human case occurrence (locations of one or more cases) per area (Figure 3) (*R*^2^ = 0.98, *P* < 0.001) increased with elevation. Mean elevation of sites surveyed that yielded at least one seropositive *P. maniculatus* was 1,612 m (range: 79–2,633 m).

### Molecular characterization.

Sin Nombre virus genetic sequences were obtained from 12 patients (13%). These sequences were compared with SNV sequences from* P. maniculatus* collected from possible putative exposure locations or other regions of the state to help corroborate suspected exposure locations. The CDPH has amassed over 300 SNV sequences from rodents collected between 2003 and 2020 that demonstrate broad geographical diversity (up to 10–15% sequence divergence across California) (CDPH, unpublished data) and provide support for determining a likely region (e.g., coastal, Northern Sierra, and Southern Sierra) of exposure for human cases. For 10 cases, these comparisons supported suspected exposure in California or in a specific geographic region of California. Sequences from two patients were most similar to sequences from *P. maniculatus* in regions of California other than where the patients resided and were discordant with available epidemiologic and environmental investigation information.

### Determinations on exposure locations and circumstances.

Both epidemiologic investigation information and environmental investigation information were used to assign a probable exposure determination (i.e., county of residence, out of county, or undetermined) to 64 (72%) of the cases (Table 1). Epidemiologic or environmental investigation information alone was used to determine an exposure location for 13 (15%) of the cases. Sin Nombre virus genetic sequences contributed to determining an exposure location for all 12 of the cases for which they were available.

**Table 1:** Source of data used to determine likely exposure locations for hantavirus cases in California

How Location Was Identified	Exposure Location (counts)			Total
	County of Residence	Out of County	Undetermined	
Epidemiological only	7	1	2	10
Environmental investigation only	3	0	0	3
Sequencing only	0	0	2	2
Epidemiological + environmental	39	21	4	64
Epidemiological + sequencing	0	0	0	0
Environmental + sequencing	1	0	0	1
Epidemiological + environmental + sequencing	6	3	0	9
Total	56	25	8	89

The patient’s county of residence was identified as the probable exposure location for 56 (63%) of the cases, whereas 25 (28%) were likely exposed in a California county (20) or state in which they did not reside (5). A likely exposure location, within or outside California, could not be determined for eight cases (9%) because the available information was insufficient or suggested multiple exposure locations (Table 1). Sin Nombre virus sequences from two patients with incomplete travel history were most similar to those from deer mice that were collected from regions outside of their counties of residence.

**Figure 4.**
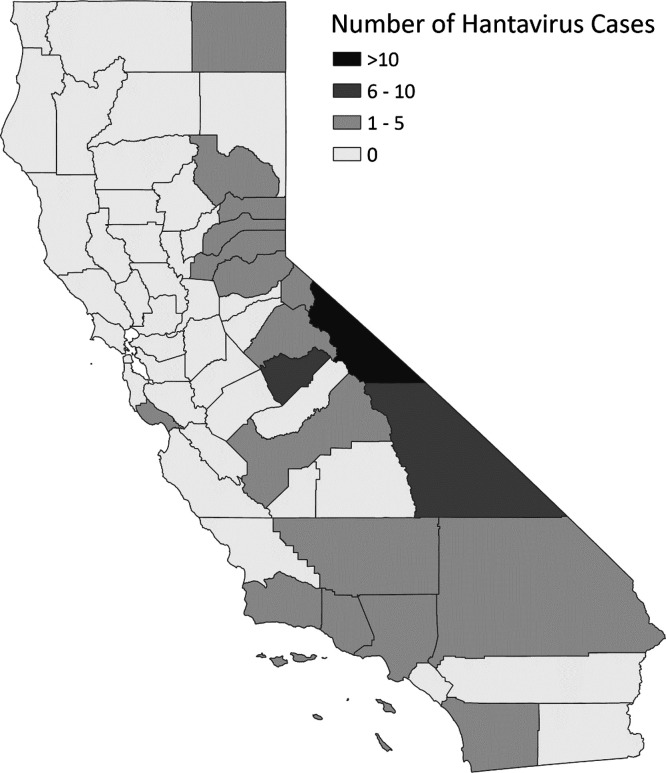
Hantavirus cases in California residents by county of likely exposure. County of exposure for 76 cases was determined by patient history, Sin Nombre virus sequencing, and epidemiologic and environmental investigations.

Nineteen (33%) of California’s 58 counties were identified as the county of exposure for at least one case. Of the 76 cases with exposure attributed to a county within the state, the greatest numbers of cases were in the Eastern Sierra and Sierra Nevada regions (Figure 4) and included 23 patients (30%) exposed in Mono County, 9 patients (12%) exposed in Inyo County, and 7 patients (9%) exposed in Mariposa County. From 1993 to 2020, the incidence of hantavirus in Mono County residents was 5.03 cases per 100,000 population, and in Inyo County, incidence was 1.4 cases per 100,000 population.

Eighty (90%) of 89 cases were isolated occurrences of known SNV infection. Multiple cases associated with the same exposure time and location were identified on two occasions. In 2012, seven cases were identified in California residents after travel to the Yosemite Valley (Mariposa County). This outbreak also included two cases in residents of other states and one case from another region (Tuolumne County) of Yosemite National Park.^17^ In 2015, two cases were likely exposed in the same residence in Mono County. In addition to these coincident cases, multiple cases with presumed exposure in the same county during the same year occurred 10 times (a total of 22 cases), with 6 of these occurrences (a total of 13 cases) in Mono County. For five (50%) of these occurrences, onset of symptoms was during the same month or consecutive months.

Sixty patients (67%) were likely exposed indoors, and two patients (2%) were likely exposed outdoors while camping; for 27 cases (30%), indoor/outdoor exposure could not be determined. Twelve patients (13%) resided in manufactured homes, whereas 15 patients (17%) resided in or had recently occupied a trailer/recreational vehicle or motor home before onset of symptoms.

**Figure 5.**
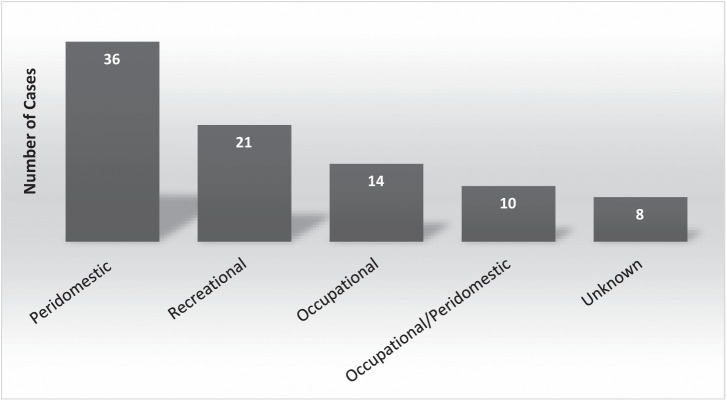
Exposure circumstances for 89 hantavirus cases in California residents.

Probable exposure circumstances were attributed as peridomestic (in or around a living space) for 36 (40%) of the cases, occupational for 14 (16%), and either occupational and/or peridomestic for an additional 10 (11%) of the cases (Figure 5). Ten cases resided full or part time at their worksite, of which eight were classified as occupational/peridomestic exposure. Twenty-one (24%) of the cases were attributed to recreational exposure. Three of the recreational exposures were possibly acquired outside of California, whereas the remaining 18 were attributed to exposure in seven counties in the Sierra Nevada (15) or in the Eastern Sierra (3). Exposure circumstances were unknown for eight cases (9%).

## DISCUSSION

Since the recognition of HPS in 1993, hantavirus cases in California residents are a relatively rare occurrence, with a median of three cases reported per year. The majority of cases were likely infected with SNV within the state and within their county of residence. Although HPS accounted for over 90% of the cases, several nonpulmonary hantavirus infections were identified. Previous studies have documented the occurrence of this clinical manifestation of SNV infection,^8^^,^^13^ but given its milder symptoms, nonpulmonary hantavirus infections may be underreported.

California cases resemble national statistics regarding major demographic and outcome measures. Sixty-three percent of the California cases were male compared with 62% of nationally reported cases,^9^^,^^34^ and the case fatality rate in California residents was similar, although slightly lower (31% versus 35%).

Reported hantavirus cases in California residents have occurred in all regions of the state except the northwestern coast. The highest number of cases occurred in the Eastern Sierra, particularly in Mono and Inyo Counties, a region with low resident population but popular recreational areas for many Californians and out-of-state visitors. Hantavirus incidence (the number of cases in residents divided by the average population in that time period multiplied by 100,000) in Mono and Inyo County residents (5.03 and 1.4 cases per 100,000 population, respectively) is hundreds of times greater than the incidence for California as a whole (0.008 cases per 100,000 population). Public health responses to hantavirus include social media posts from CDPH in late spring when the risk of encountering hantavirus is greatest as cabins and other closed spaces are first being opened for the season. In addition, presentations on awareness and diagnostic approaches, such as using the five-point screening method, are provided to the local medical community.^35^

Although deer mice can be found throughout the state, their abundance and SNV infection prevalence vary regionally,^19^ and SNV prevalence in deer mice is typically greater at higher elevations.^22^^,^^36^ Correspondingly, we found that the incidence of hantavirus infection increased in higher elevation areas; more than three quarters of hantavirus cases were likely exposed at elevations from 1,000 to 2,499 m. No cases were determined to be from above 2,700 m (8,858 feet), which is likely because of the relative paucity of inhabited structures at or above this elevation rather than the scarcity of infected deer mice. Environmental factors, SNV prevalence in deer mouse populations, and deer mouse abundance in proximity to humans, particularly within structures, may contribute to the regional differences in the incidence of human infections.^22^^,^^37^

Our findings support the recognized link between direct contact with deer mice and other activities that aerosolize infective virus in enclosed spaces, which results in transmission of SNV to humans. Forty percent of patients or their proxies reported contact with rodents or cleaning or related activities in enclosed structures with evidence of rodent activity, and a majority reported seeing evidence of recent rodent activity at their residence or worksite. Almost all of our environmental investigations where rodent trapping was conducted found at least one deer mouse, and at least one seropositive deer mouse was found in over two thirds of the investigations. The common finding of SNV-positive deer mice at the locations investigated is not surprising but was significantly higher than routine surveillance (20% versus 11%) conducted in the state during the same period. These findings did help support the determination of likely exposure locations in many cases, and they presented an opportunity to provide public health information to prevent future exposure.

No rodent species other than *P. maniculatus* have been implicated in the transmission of SNV to humans in California. High SNV prevalence has been observed in *Peromyscus eremicus* where the sympatric deer mouse population appeared to be low, suggesting that this species and others are also capable of maintaining the virus.^27^^,^^38^ Similarly, SNV sequences obtained from multiple rodent species in New Mexico suggest that secondary transmission risks from other rodents in the western United States should not be dismissed without additional investigation.^39^^,^^40^

Similar to other studies,^6^^,^^9^ we identified that case exposure most commonly occurs in and around living spaces (i.e., peridomestic). Although recreational exposures were more numerous than occupational exposures, this comparison was biased by the eight recreational exposures (38%) associated with the unusual 2012 outbreak in Yosemite National Park, including seven patients who lodged in Yosemite Valley guest cabins. Furthermore, we could not differentiate occupational or peridomestic exposure for a substantial number of cases. Instead, we classified them as occupational/peridomestic, notably because 8 of these 10 individuals resided at their worksites. All workplace residences for which we had information had reports of rodent activity. Worksite lodgings may be an underappreciated work-related hantavirus risk and a point of emphasis for occupational risk training when these situations exist.

Manufactured homes or nonpermanent living structures (motorhomes, recreation vehicles, or trailers) that had been occupied in the 2 months before disease onset were identified as likely exposure locations for 30% of cases. Such domiciles may be more common in rural or mountainous habitats where deer mouse populations are often abundant. They may have more potential rodent access points than traditional residences on permanent foundations, and some combination of these and related factors may present greater opportunities for rodent ingress. Focused educational efforts on habitat modification and rodent exclusion in and around these structures should be considered, particularly in rural environments and for transient recreationalists who may not be aware of regional disease risks.

Although environmental investigation and molecular characterization of SNV sequences can be resource intensive, these efforts have proven useful in identifying likely exposure locations and circumstances and providing important prevention information. Most often, information from both epidemiologic and environmental investigations was helpful for making these determinations. In many instances, the on-site environmental investigation was important in identifying specific risk situations and conveying mitigation measures to homeowners, employers, or recreational management to decrease risk for additional cases.^9^^,^^21^^,^^41^ For example, the environmental follow-up of the 2012 Yosemite outbreak^17^ yielded insight into necessary changes to guest cabin structures and rodent control practices, underscoring the value of environmental follow-up of hantavirus cases.

Sequence data to compare SNV isolates from human cases and rodents have been used for many years to support identification of likely exposure locations.^42^ The utility is increasing as more detailed sequence characterizations become available, and there is greater documentation of regional SNV variants in deer mouse populations. Phylogenetic analyses of over 300 rodent SNV sequences sampled from multiple locations across 20 California counties show high genetic diversity (up to 15% sequence divergence) of the virus and have revealed enough geographic variation to provide support for a likely region of human case exposure in many case investigations. As an example, one recent CDPH VBDS investigation involved a California patient who resided in a coastal county but had traveled to mountainous regions both in and out of state in the months preceding symptom onset. The patient reported observing mouse droppings in a cabin in Montana but not in a cabin or while camping in the Sierra Nevada. The follow-up investigation did not find evidence of rodent activity in the California cabin, suggesting that the patient was exposed in Montana. However, the SNV isolate obtained from the patient was dissimilar to SNV sequences from Montana and highly similar to SNV sequences from deer mice previously collected from the northern Sierra Nevada region where the patient recreated (CDPH, unpublished data). This case investigation highlights the importance of collaborating with other agencies and laboratories with sequencing capability.

The CDPH VBDS was either the lead agency or assisted with >90% of the case investigations and has used findings from these investigations to better inform Californians and visitors of the risks associated with hantavirus exposure. Educational materials have been created to support hantavirus awareness and infection prevention in California, especially among at-risk populations who live, work, or recreate in areas where infected deer mice may be present.^19^ These include an interactive story map developed to provide the public with general information, cases by county, approximate locations of deer mouse collections, and antibody seroprevalence by county. Since 2014, CDPH VBDS has offered a standardized facility evaluation for reducing hantavirus risks to agencies (e.g., U.S. Forest Service, National Park Service, and California State Parks) with employees working or living in areas at high risk for hantavirus infection. The evaluation is a tool to assist employees in identifying and mitigating existing or potential rodent infestation issues. The facility evaluation focuses on known risk factors (e.g., presence of or potential for deer mouse infestation) that can be directly observed and evaluated.

## CONCLUSION

Our findings demonstrate the utility of a multifaceted investigative approach and emphasize the value of conducting follow-up environmental investigations, including rodent trapping at sites where hantavirus cases lived or recreated. Such investigations identified rodent activity or confirmed deer mouse presence at many sites, subsequently prompting mitigation efforts. Integrating epidemiologic information and molecular evidence with findings from the environmental investigations has proven useful in determining likely location and circumstances of case exposures, preventing additional exposures, and improving our knowledge of hantavirus epidemiology in California.

## Supplemental Materials

10.4269/ajtmh.25-0270 Supplemental Materials
