# Supplementary material for: Epidemiologic and Environmental Investigations of Reported Hantavirus Cases Inform Exposure Risk in California, 1993–2020
Source: Am J Trop Med Hyg. 2025 Oct 16;113(6):1385–92. doi: 10.4269/ajtmh.25-0270 (PMC12676593; doi:10.4269/ajtmh.25-0270)
Supplement: Supplemental Materials [file tpmd250270.SD1.pdf]

Supplemental Materials

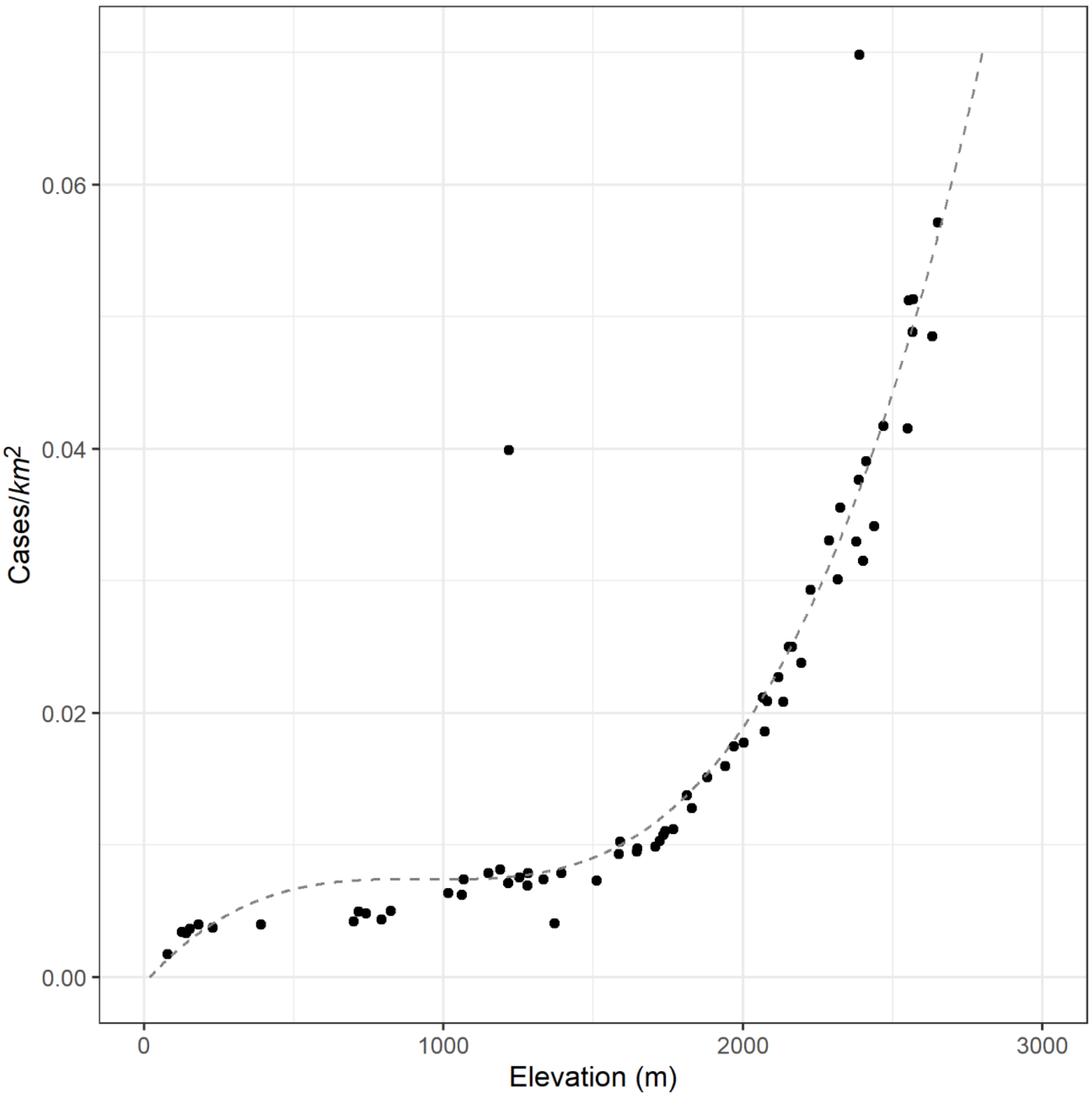

Figure 1. Hantavirus cases/km^2 by elevation. Fitted line is from best fitting polynomial regression model (R^2 = 0.085, P <0.001).

Table 1

AIC comparison of models predicting hantavirus cases by elevation

Models ~ Hantavirus Cases	AIC	ΔAIC	k	AIC Weight
elevation+elevation^2+elevation^3	-476.35	0	4	0.72
elevation+elevation^2+elevation^3+elevation^4	-474.48	1.87	5	0.28
elevation+elevation^2	-461.46	14.88	3	0
elevation	-417.89	58.46	2	0

Table 2

AIC comparison of models predicting occurrences of hantavirus cases by elevation

Models ~ Hantavirus Occurrences	AIC	ΔAIC	k	AIC Weight
elevation+elevation^2+elevation^3	-631.68	0	4	0.51
elevation+elevation^2+elevation^3+elevation^4	-631.56	0.11	5	0.49
elevation+elevation^2	-553.49	78.19	3	0
elevation	-446.81	184.87	2	0
